# Clarifying the relationship between mindfulness and executive attention: a combined behavioral and neurophysiological study

**DOI:** 10.1093/scan/nsy113

**Published:** 2018-12-11

**Authors:** Yanli Lin, Megan E Fisher, Jason S Moser

**Affiliations:** Department of Psychology, Michigan State University, East Lansing, MI, USA

**Keywords:** mindfulness, executive attention, P3, congruency effects

## Abstract

Mindfulness is frequently associated with improved attention. However, the nature of the relationship between mindfulness and executive attention, a core function of the attentional system, is surprisingly unclear. Studies employing behavioral measures of executive attention have been equivocal. Although neuroscientific studies have yielded more consistent findings, reporting functional and structural changes in executive attention brain regions, the observed changes in brain activity have not been linked to behavioral performance. The current study aimed to fill these gaps in the literature by examining the extent to which trait mindfulness related to behavioral and neurophysiological (indexed by the stimulus-locked P3) measures of executive attention. Results revealed that higher trait mindfulness was related to less flanker interference on accuracy and reaction time, consistent with enhanced executive attention. Critically, mediational analyses showed that the P3 accounted for the relationship between trait mindfulness and executive attention performance, elucidating a neural mechanism through which mindfulness enhances executive attention.

## Introduction

Mindfulness has received accelerated interest from various sectors of society, with media, corporations and schools touting the benefits of mindfulness for work productivity, academic performance and other cognitively demanding tasks (Boyce, 2011). In response to this surging popularity, researchers have cautioned that public enthusiasm for mindfulness may be outpacing scientific progress (Van Dam *et al.*, 2018). Indeed, mindfulness is a relatively nascent topic of scientific inquiry, and although substantial work has been devoted to understanding the effects of mindfulness, far less is known about how it works. More than ever, concerted efforts aimed at elucidating the underlying mechanisms of mindfulness are needed to both understand and validate its effects—ultimately, to determine the extent to which the *‘*hype*’* is warranted.

Part of the impetus behind the *‘*mainstreaming*’* of mindfulness is a growing scientific literature supporting its benefits to attention (see Chiesa *et al.*^2011^; Hӧlzel *et al.*, 2011; Tang *et al.*, 2015, for reviews). Theoretically, attention is widely regarded as a core aspect of mindfulness that cuts across its different aspects as a polylithic psychological construct (e.g. state, trait, meditative practice and therapeutic intervention; Brown and Ryan, 2003; Bishop *et al.*, 2004; Shapiro *et al.*, 2006; Hӧlzel *et al.*, 2011; Vago and Silbersweig, 2012; Tang *et al.*, 2015). For example, mindfulness meditation typically involves moment-to-moment application, sustainment and redirection (in the event of distraction) of attention to a target object (Lutz *et al.*, 2008). Consistent practice over time has been associated with trait-like changes in attentional brain networks, ostensibly constituting a neural mechanism through which the abilities cultivated during training transfer *‘*off the cushion*’* into daily life (Hasenkamp and Barsalou, 2012). Consequently, understanding the nature of the relationship between mindfulness and attention appears pertinent for elucidating the mechanisms involved in mindfulness and its seemingly far-reaching impact on a variety of life functions (e.g. occupational performance).

Reviews have generally associated mindfulness with improved attention (Chiesa *et al.*, 2011; Hӧlzel *et al.*, 2011; Vago and Silbersweig, 2012; Tang *et al.*, 2015). Attention, like mindfulness, however, is a complex multidimensional construct that is often divided into three distinct but interrelated networks (Petersen and Posner, 2012): (i) alerting, defined as readiness or preparation for an impending stimulus; (ii) orienting, defined as the selective targeting of specific sensory input based on modality or location; and (iii) executive attention (also referred to as conflict monitoring), defined as the goal-directed monitoring of task-relevant stimuli in the midst of competing task-irrelevant stimuli. A closer examination of the literature at the subnetwork level reveals a more nuanced state of affairs.

With regard to alerting, short-term training studies have not yielded significant effects (Jha *et al.*, 2007; Tang *et al.*, 2007), whereas long-term mindfulness training and intensive meditative experience appear to relate to improved alerting (Pagnoni and Cekic, 2007; MacLean *et al.*, 2010). Improvements in orienting, on the other hand, have been reported in both short- and long-term training studies (Jha *et al.*, 2007; MacLean *et al.*, 2010), in addition to cross-sectional studies comparing meditators with controls (van den Hurk *et al.*, 2010).

Interestingly, studies on the relationship between executive attention and mindfulness have been the most equivocal and inconclusive. Of the five prospective training studies reviewed in Chiesa *et al.* (2011), three studies reported positive outcomes (Tang *et al.*, 2007; Wenk-Sormaz, 2005; Jha *et al.*, 2007), but the other two reported no change (Polak, 2009; Anderson *et al.*, 2007). More recent randomized controlled studies are also mixed. Semple (2010) and Josefsson *et al.* (2014) did not detect changes in behavioral measures of executive attention, whereas Smart and Segalowitz (2017) reported faster reaction times following mindfulness training—suggestive of enhanced performance efficiency. Cross-sectional studies are similarly equivocal, with Chan and Woollacott (2007), Moore and Malinowski (2009) and Van den Hurk *et al.* (2010) reporting positive correlations between mindfulness meditation experience, self-reported trait mindfulness and executive attention. On the other hand, Josefsson and Broberg (2011) and Lykins *et al.* (2012) observed no differences in performance between meditators and controls, and Schmertz (2006) found no significant correlations between self-reported mindfulness and executive attention.

Highlighting the extent of the ambiguity surrounding the mindfulness–executive attention relationship, Josefsson *et al.* (2014) have called to remove executive attention from theoretical models of mindfulness. However, such strong claims are challenged by the fact that executive attention is widely conceptualized to be an integral component of mindfulness meditation itself (see Vago and Silbersweig, 2012; Hӧlzel *et al.*, 2011). Consequently, it stands to reason that the recruitment of executive attention during mindfulness meditation may engender its *‘*off-the-cushion*’* effects on behavioral measures of executive attention—illustrating the porous boundaries between process and effect. In addition, mindfulness meditation is only one of the many aspects of mindfulness—examining how other aspects of mindfulness relate to executive attention is pertinent to developing a more holistic understanding of the broader relationship. Given these considerations, advocating removal of executive attention from theoretical frameworks of mindfulness solely on grounds of the inconsistency to detect behavioral changes after brief mindfulness training appears premature and unwarranted.

Indeed, neuroimaging research appears to support the existence of a relationship between mindfulness and executive attention (Tang *et al.*, 2015). Specifically, Tang *et al.* (2015) noted that mindfulness training studies of attention have most consistently detected changes in the anterior cingulate cortex (ACC)—a region that has been extensively implicated in executive attention (Botvinick *et al.*, 2004; Bush *et al.*, 2000; Posner *et al.*, 2007; Peterson and Posner, 2012). Cross-sectional designs yield similar findings, reporting enhanced ACC activation (Hӧlzel *et al.*, 2007; Gard *et al.*, 2012) and cortical thickness (Grant *et al.*, 2010) in experienced meditators relative to novice controls. Moreover, other longitudinal training studies have found activation changes in the prefrontal cortex (Allen *et al.*, 2012; Hӧlzel *et al.*, 2013; Zeidan *et al.*, 2013), another key region of executive attention (Kane and Engle, 2002).

Although these studies have yielded considerable evidence supporting mindfulness-related changes in executive attention brain regions, crucial questions remain about whether or how these changes relate to behavioral measures of executive attention. Further complicating matters is that, as summarized above, behavioral measures of executive attention have not been reliably linked to mindfulness. We view these issues as a by-product of two long-standing interrelated challenges associated with neurocognitive mindfulness research. First, it is difficult to obtain precise measures of the interplay between brain and behavior. For example, the oft-discussed temporal constraints of functional magnetic resonance imaging preclude a time-sensitive mapping between mindfulness-related changes in regional activation and online task performance. Second, this limitation is compounded by the polylithic nature of mindfulness as a construct, such that reported effects (or lack thereof) may be confounded by a variety of idiographic (e.g. trait mindfulness and meditative experience), training (e.g. didactics, practice frequency and duration) and task-related confounds (e.g. engaging in state mindfulness during a *‘*non-mindful*’* task condition). That is, in order to obtain consistent findings and draw meaningful conclusions about the nature of the mindfulness–executive attention relationship, it may be prudent for studies to examine the interplay between brain and behavior while accounting for confounds associated with construct heterogeneity. Consequently, a reasonable first step may be to examine the extent to which trait mindfulness, in a meditation-naïve sample, relates to behavioral and neural indices of executive attention. This approach leverages natural variability in trait mindfulness while minimizing confounds associated with the other aspects of mindfulness (e.g. meditative experience).

Indeed, the purpose of the current study was to explore the relationship between trait mindfulness and executive attention by utilizing a combined behavioral and neurophysiological paradigm. Toward this end, we leveraged the versatility and temporal precision of electroencephalography to record neural activity during performance of the Eriksen Flankers task (Eriksen and Eriksen, 1974), a classic executive attention task (Rueda *et al.*, 2005). In the flanker task, the task-relevant(target) and task-irrelevant (flankers) stimuli either elicit the same response (congruent) or opposite responses (incongruent). A primary axiom is that executive attention is needed to resolve competing response representations elicited from the flanking arrows. Consequently, behavioral indices of executive attention are typically computed as the relative performance [i.e. accuracy and response time (RT)] between incongruent and congruent trials (Fan *et al.*, 2002)—with larger differences between performance on incongruent and congruent trials, i.e. more errors and longer RTs on incongruent relative to congruent trials) reflecting poorer executive attention ability.

In addition to behavioral performance measures, we examined the stimulus-locked P3, an event-related potential (ERP) thought to broadly reflect attention processing (Polich, 2007; Clayson and Larson, 2011; Frühholz *et al.*, 2011; Groom and Cragg, 2015). The P3 is a centro-parietal positive deflection that peaks 350–650 ms after stimulus presentation and is larger for incongruent relative to congruent trials (Kopp *et al.*, 1996; Frühholz *et al.*, 2011; Hillman *et al.*, 2009). Source localization work has linked the flanker P3 to inhibitory control regions (i.e. inferior frontal cortex; Frühholz *et al.*, 2011; Nee *et al.*, 2007) suggesting that the enhanced P3 elicited by incongruent *vs* congruent stimuli reflects attentional inhibition of the incongruous flanker stimuli. Alternatively, others have proposed that the P3 reflects recruitment of attentional resources in response to increased demand for cognitive control (Clayson and Larson, 2011). Despite these differences in functional conceptualization, there is a consensus that the P3 is sensitive to the difference in attention processing between incongruent and congruent stimuli. Consequently, the P3 elegantly compliments behavioral measures of executive attention insofar that both neural and behavioral operationalizations of executive attention reflect the difference between incongruent and congruent stimuli. Another advantage of the P3 involves its unique temporal position within the broader process of flanker task performance, such that it reflects online attention processing of flanker stimuli prior to the behavioral response. Therefore, measurement of the P3 allows the drawing of process-oriented inferences regarding executive attention—namely, how neural processing influences subsequent behavioral performance.

Our study, as part of a secondary data analysis of a randomized controlled trial, was therefore designed to explore the relationship between trait mindfulness and both behavioral and neural indices of executive attention. Given the mixed findings from behavioral paradigms and the notable absence of studies involving simultaneous measurement of brain and behavior, we established our expectations on theoretical grounds. Executive attention is defined as the conscious recognition of an object in service of goal fulfillment (Posner and Raichle, 1994; Rueda *et al.*, 2005). Consequently, conscious awareness, also referred to as focal attention, is required to discriminate goal-relevant targets from competing goal-irrelevant stimuli (Peterson and Posner, 2012). During behavioral tasks of executive attention (e.g. flanker task), sustained moment-to-moment recruitment of focal attention is necessary to maintain task performance. In this light, executive attention exhibits considerable theoretical overlap with mindfulness, such that present moment conscious awareness cuts across both constructs. During mindfulness meditation, present moment awareness is directed toward arising internal experience in the midst of cognitive distraction (e.g. rumination and elaboration); during the flanker task, awareness is repeatedly directed toward the central target stimulus in the midst of flanking stimuli. As such, we advance the position that executive attention is a core aspect of mindfulness that cuts across its different facets. Not only is executive attention recruited during mindfulness training and possibly strengthened as an effect of training, but individuals with higher levels of trait mindfulness are also expected to possess superior executive attention ability relative to those who are less dispositionally mindful.

One critical caveat, however, is that trait mindfulness is itself a diverse, polylithic construct and its measurement subject to considerable debate (Grossman, 2011). In response to the varied semantic definitions of mindfulness and accompanying methodological challenges, the Five-Factor Mindfulness Questionnaire (FFMQ; Baer *et al.*, 2006) was developed to capture the different facets of trait mindfulness: observing, describing, acting with awareness, non-judging and non-reactivity. Among these facets, the acting with awareness subscale (FFMQ-AA) measures the propensity to attend to the present moment. Given our theoretical framework, the FFMQ-AA, or what we deem trait mindful awareness, appears to be the most relevant facet of trait mindfulness in the broader mindfulness–executive attention relationship. Specifically, items on the FFMQ-AA pertain to the degree of attentional awareness in everyday life (e.g. ‘It seems I am “running on automatic” without much awareness of what I’m doing’), as opposed to technical abilities or personal qualities developed through contemplative training (Brown and Cordon, 2009; Goodman *et al.*, 2015). Equally important, the FFMQ-AA has been shown to relate to neural measures of motivated attention (Brown *et al.*, 2012; Lin *et al.*, 2016) and behavioral measures of executive attention (Di Francesco *et al.*, 2017), demonstrating its sensitivity to multimodal measures of attention.

Therefore, we expected that trait mindful awareness would be related to improved behavioral and neural measures of executive attention. Specifically, trait mindful awareness should be related to smaller flanker interference effects on RTs and accuracy, reflecting superior executive attention in more dispositionally mindful individuals (Fan *et al.*, 2005). Neurally, we reasoned that trait mindful awareness would likewise relate to a smaller difference in P3 amplitude between incongruent and congruent stimuli. Leveraging the full potential of our multimodal design, we further explored the interplay between brain and behavior by testing whether the P3 mediated the observed relationships between trait mindful awareness and behavioral performance.

Lastly, as discussed above, measurement of trait mindfulness remains a significant challenge to mindfulness research. Although trait mindful awareness (FFMQ-AA) appears most theoretically relevant, investigating relationships between the other facets of trait mindfulness and executive attention appears prudent. Toward this end, we elected to explore the relationships between the other four facets of the FFMQ and behavioral and neural measures of executive attention.

## Method

### Participants

Sixty-three native English-speaking, right-handed, female undergraduates completed the current measures as part of a larger randomized controlled trial examining the effects of mindfulness training on gender stereotype threat. The current study involved data collected prior to assignment of the intervention conditions. Thus, all participants were naïve to mindfulness training and did not differ in their exposure to the interventions or task conditions. We recruited female participants to address research questions related to gender stereotype threat. An all-female sample also minimizes confounds related to gender differences in P3 amplitudes (see Polich and Martin, 1992; Hoffman and Polich, 1999). Furthermore, there are emerging studies reporting gender differences in responsivity to mindfulness meditation (de Vibe *et al.*, 2013; Luders *et al.*, 2015). Although the present study did not test effects of mindfulness training, an all-female sample could minimize potential gender-related confounds in the broader mindfulness–executive attention relationship. Three participants were excluded from the analyses because of a failure to follow instructions regarding the stimulus-response mapping (see below) that resulted in an error rate exceeding 50%. The final sample consisted of 60 participants. Psychopathology and medication use were not assessed for inclusion in the study. No participants discontinued their involvement after consenting.

### Flanker task

Participants completed an arrow version of the Eriksen flankers task (Eriksen and Eriksen, 1974). Participants were seated ~60 cm in front of a computer monitor and instructed to respond to the center arrow of a five-arrow string in which the target was either congruent (e.g. <<<<< or >>>>>) or incongruent (e.g. <<><< or >><>>) with the surrounding (i.e. flanking) arrows. Characters were displayed in a standard white font on a black background and subtended 1.3 degrees of visual angle vertically and 9.2 degrees horizontally. The task was administered on a Pentium R Dual Core computer using E-Prime software (Psychology Software Tools, Inc. Sharpsburg, PA 15215-2821 USA), which facilitated stimuli presentation and response measurement.

During each trial, arrows were presented for 200 ms. Participants were given 950 ms to respond before the next intertrial interval began. During the intertrial interval, a fixation cross (+) was presented and varied in duration between 600 and 1000 ms. The experimental session included 512 trials grouped into 8 blocks of 64 trials. Half of the trials in each block were congruent and half were incongruent. Participants were instructed to respond as quickly and accurately as possible using either their right index or middle finger to select the left and right mouse buttons, respectively, that corresponded with the target direction. To encourage speed, accuracy and sufficient error commission, performance-based feedback was presented at the end of each block. If performance accuracy fell below 75%, participants were instructed to respond more accurately. When performance was above 90%, participants were instructed to respond faster. Accuracy in between 75% and 90% elicited the feedback *‘*You’re doing a great job*’*.

### Trait mindfulness

Trait mindful awareness was measured by scores on the acting with awareness subscale on the FFMQ (Baer *et al.*, 2006). Scores on the other four facets were used to measure (i) observing (FFMQ-O), defined as noticing internal and external experiences; (ii) describing (FFMQ-D), defined as verbal articulation of internal experiences; (iii) non-judging (FFMQ-NJ), defined as adopting a non-evaluative perspective toward thoughts and feelings; and (iii) non-reactivity (FFMQ-NR), defined as allowing inner experiences to pass without attachment or elaboration. Participants responded to the 39-item subscale using a 5-point Likert scale ranging from 1 (never or very rarely true) to 5 (very often or always true).

### Psychophysiological recording and data reduction

Participants were fitted with a 64-channel stretch-lycra cap. Continuous electroencephalographic activity was recorded using the BioSemi ActiveTwo system (BioSemi, Amsterdam, the Netherlands). Recordings were taken from 64 Ag-AgCl electrodes placed in accordance with the 10/20 system. Two additional electrodes were placed on the left and right mastoids. Electrooculogram activity generated by eye movements and blinks was recorded at FP1 and at three electrodes placed inferior to the left pupil and on the left and right outer canthi ~1 cm from the pupil. During data acquisition, the common mode sense active electrode and driven right leg passive electrode formed the ground, as per BioSemi’s design specifications. All signals were digitized at 1024 Hz.

**Fig. 1:**
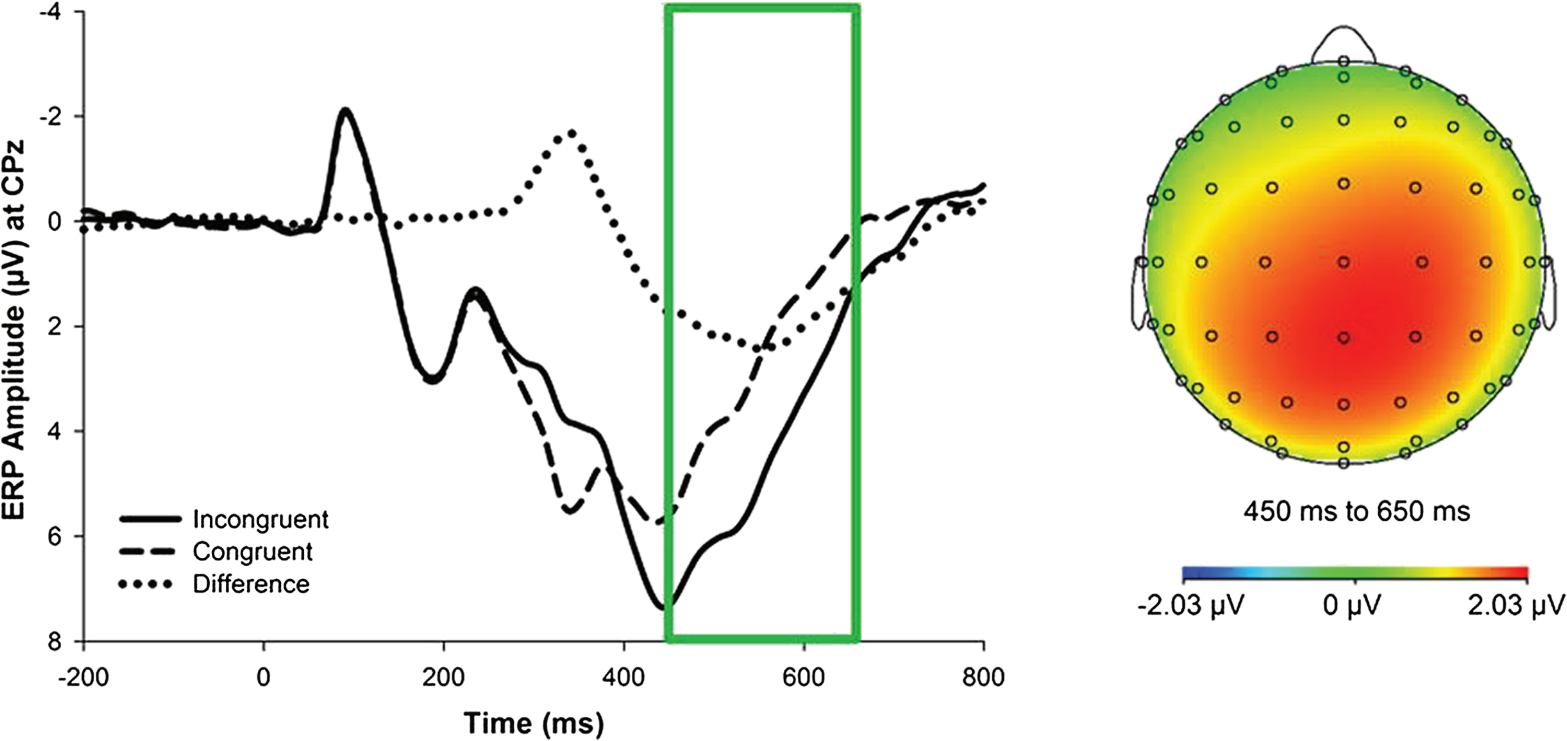
Left: stimulus-locked, grand average waveforms depicting congruent P3, incongruent P3 and ΔP3 amplitudes at electrode CPz. Time 0 is the stimulus onset. Right: scalp topography depicting voltages of the ΔP3, quantified as the average activity between 450 and 650 ms following stimulus onset.

Offline analyses were conducted using BrainVision Analyzer 2 (BrainProducts, Gilching, Germany). Scalp electrode recordings were referenced to the numeric mean of the mastoids and band-pass filtered with cutoffs of 0.1 and 30 Hz (12 dB/oct roll-off). Ocular artifacts were corrected using the regression method developed by Gratton *et al.*, (1983). Physiological artifacts were detected using a computer-based algorithm such that trials in which the following criteria were met were rejected: a voltage step exceeding 50 μV between sampling points, a voltage difference of >300 μV within 200 ms intervals, voltage exceeding ±200 μV or a maximum voltage difference <0.5 μV within 100 ms intervals. ERPs were locked to stimuli (i.e. flankers) onset, with a 200 ms pretrial baseline correction. In accordance with the literature, only correct responses were included in the final average for P3 measurement (Frühholz *et al.*, 2011; Clayson and Larson, 2011; Rietdijk *et al.*, 2014). In line with the collapsed localizer method (Luck and Gaspelin, 2017), the P3 was then quantified as the average activity occurring between 450 and 650 ms post-stimulus onset at the centro-parietal recording site CPz—where the amplitude was maximal (Figure 1). A voltage map for the incongruent–congruent difference wave for the P3 is also presented in Figure 1.

### Overview of analyses

Behavioral and ERP measures were statistically analyzed using SPSS software (version 23.0). Specifically, behavioral data were submitted to repeated measures analyses of variance (rANOVAs) and paired *t*-tests. Partial eta squared (${\eta}_p^2$) is reported as an estimate of effect size in ANOVA models where 0.05 represents a small effect, 0.1 a medium effect and 0.2 a large effect (Cohen, 1973). Overall numbers of errors were submitted to a paired *t*-test to compare performance accuracy as a function of stimulus type (congruent *vs* incongruent). Overall RTs were submitted to a two stimulus-type (congruent *vs* incongruent)–two accuracy (correct *vs* error) rANOVA. When significant interactions emerged, follow-up *t*-tests were conducted to aid in the interpretation of results. Degrees of freedom varied slightly among the F-tests because of performance variability (e.g. a participant making no errors on congruent trials is excluded from analyses involving congruency). As is standard, congruency effects on accuracy and RTs were computed as the difference in errors and difference in correct response RTs between incongruent and congruent trials, respectively. For ERP analyses, a paired *t*-test was used to compare P3 amplitudes as a function of stimulus type (congruent *vs* incongruent) to confirm the well-established effect that P3 amplitudes are larger on incongruent relative to congruent trials. In subsequent analyses, ΔP3 was quantified as the difference in amplitude between incongruent and congruent stimuli.

To achieve the primary aim of the current study, correlational analyses involving associations among FFMQ-AA, behavioral performance measures (i.e. congruency interference effects on error rate and RTs) and ΔP3 were conducted to determine the extent to which trait mindfulness related to behavioral and neural measures of executive attention. Moreover, two mediation models were tested to examine whether online neural processing (ΔP3) mediated the expected relationships between mindfulness and behavior. In both models, FFMQ-AA scores were entered as the independent variable and ΔP3 as the mediator. The two models only differed in the dependent variable, such that the first involved the congruency difference in errors (i.e. incongruent–congruent errors), whereas the second involved the congruency difference in RT (i.e. incongruent–congruent RTs). The analyses were performed using the SPSS macro from Preacher and Hayes (2008). In accordance with the recommendations outlined in Preacher and Hayes (2004), a bootstrapping procedure involving 5000 samples with a 0.95 confidence interval (CI) was used to test indirect effects. To explore the relationships between executive attention and the other facets of trait mindfulness, the analyses were repeated separately for FFMQ-O, FFMQ-D, FFMQ-NJ and FFMQ-NR.

## Results

Descriptive statistics for behavioral, ERP and FFMQ are presented in Table 1.

**Table 1:** Summary of behavioral and ERP measures

Variable	M	s.d.
Number of errors	59.13	21.79
Number of corrects	445.40	24.43
Incongruent errors	43.67	17.23
Congruent errors	15.47	8.45
Δ Errors	28.20	16.17
Error RT (ms)	335.64	56.34
Correct RT (ms)	421.22	44.38
Incongruent error RT (ms)	340.61	64.48
Incongruent correct RT (ms)	451.12	46.68
Congruent error RT (ms)	321.55	52.99
Congruent correct RT (ms)	395.35	43.39
Δ RT (ms)	55.78	17.98
Incongruent P3 amplitude (μV)	4.74	2.58
Congruent P3 amplitude (μV)	2.71	2.25
Δ P3 amplitude (μV)	2.03	1.66
FFMQ-AA	26.23	5.93
FFMQ-O	26.80	5.41
FFMQ-D	27.69	7.91
FFMQ-NJ	26.95	5.88
FFMQ-NR	19.73	4.34

**Note:** Δ Errors, Δ RT and Δ P3 denote the difference in errors, RT and P3 amplitude, respectively, between incongruent and congruent trials.

### Establishing basic effects of behavioral and ERP measures of executive attention

Overall flanker task accuracy was relatively high (*M* percent correct = 87.47%, s.d. = 4.60%). As expected, participants made an average of 59.13 errors (s.d. = 21.79), with more errors on incongruent trials (*M* = 43.67, s.d. = 17.23) than congruent trials [*M* = 15.47, s.d. = 8.45, *t*(59) = 13.51, *P* < 0.01, *d* = 1.96].

The analysis of s.d.s revealed main effects of accuracy and congruency, such that RTs on error trials (*M* = 335.64, s.d. = 56.34) and congruent trials (*M* = 390.73, s.d. = 43.15) were faster than on correct [*M* = 421.22, s.d. = 44.38, *F*(1, 59) = 673.40, *P* < 0.01, ${\eta}_p^2$=0.92] and incongruent trials [*M* = 431.89, s.d. = 50.04, *F*(1, 59) = 94.15, *P* < 0.01, ${\eta}_p^2$ = 0.62], consistent with known speed-accuracy and speed-congruency trade-offs. These main effects were qualified by a significant accuracy–congruency interaction [*F*(1, 59) = 24.64, *P* < 0.01, ${\eta}_p^2$ = 0.30], such that RT differences between incongruent and congruent trials were larger on correct trials (*M* = 55.78, s.d. = 17.97) relative to error trials [*M* = 19.05, s.d. = 55.71, *t*(59) = 4.96, *P* < 0.01, *d* = .87].

As expected, P3 amplitudes were larger on incongruent trials (M = 4.74, s.d. = 2.58) relative to congruent trials [*M* = 2.71, s.d. = 2.25*, t*(59) = 9.48, *P* < 0.01, *d* = 0.83; see Figure 1].

In summary, behavioral and ERP measures reflected the expected effects of executive attention in the flanker task (i.e. stimulus congruency), such that a greater number of errors, slower RTs and a larger P3 characterized incongruent stimuli.

### Relationships between behavioral performance and trait mindful awareness

**Fig. 2:**
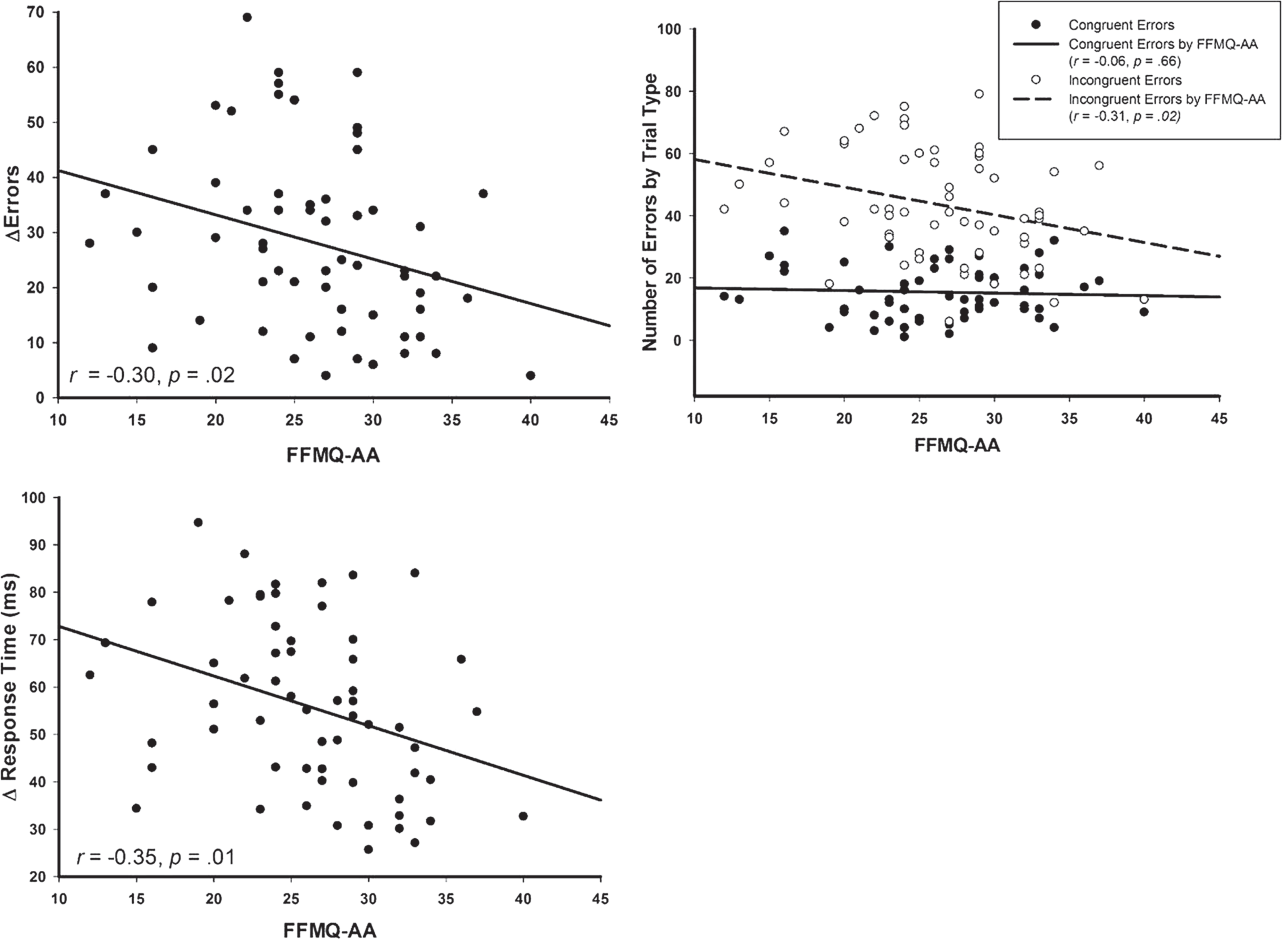
Scatterplots depicting behavioral performance as a function of trait mindfulness (FFMQ-AA). Top left: significant negative correlation between FFMQ-AA and number of errors elicited on incongruent trials (dashed line), but not congruent trials (solid line). As FFMQ-AA increases, number of errors committed on incongruent trials decreases. Top right: negative correlation between congruency difference (incongruent–congruent) in errors (Δ errors) and FFMQ-AA, such that as FFMQ-AA increases, Δ errors decreases. Bottom left: negative correlation between congruency difference (incongruent–congruent) in response time (Δ RT) and FFMQ-AA, such that as FFMQ-AA increases, Δ RT decreases.

Consistent with expectations, higher trait mindful awareness was associated with better executive attention such that higher FFMQ-AA scores were associated with a smaller incongruent–congruent difference in errors (*r* = −0.30, *P* = 0.02) and RTs (*r* = −0.35, *P* = 0.01). Follow-up analysis demonstrated that these relationships were primarily driven by performance on incongruent trials such that high FFMQ-AA participants committed fewer errors (*r* = −0.30, *P* = 0.02) and were faster (*r* = −0.35, *P* = 0.01) on incongruent trials relative to lower FFMQ-AA participants. See Figure 2 for scatter plots of the reported correlations.

### Relationships between P3 amplitude and trait mindful awareness

**Fig. 3:**
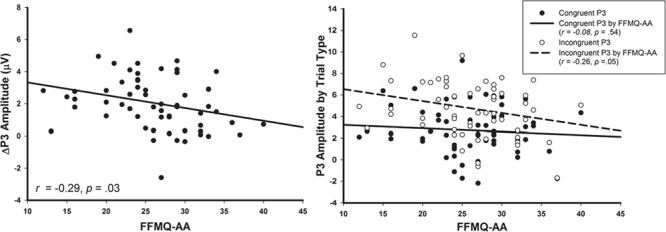
Scatterplots depicting P3 amplitude as a function of trait mindfulness (FFMQ-AA). Left: negative correlation between congruency difference (incongruent–congruent) in P3 amplitude (ΔP3) and FFMQ-AA, such that as FFMQ-AA increases, ΔP3 decreases. Right: significant negative correlation detected between FFMQ-AA and P3 amplitudes elicited on incongruent (dotted line), but not congruent trials (solid line). As FFMQ-AA increases, P3 amplitudes elicited on incongruent trials decreases.

Furthermore, we found a significant negative relationship between trait mindful awareness and the ΔP3, such that higher FFMQ-AA scores were associated with smaller ΔP3 amplitudes (*r* = −0.29, *P* = 0.03). Follow-up analysis revealed that this relationship was driven by higher FFMQ-AA scores relating to smaller P3 amplitudes on incongruent trials (*r* = −0.26, *P* = 0.05), but not congruent trials (*r* = −0.08, *P* = 0.54). See Figure 3 for scatter plots of the reported correlations.

### The mediating role of the ΔP3 in the relationship between trait mindful awareness and performance

**Fig. 4:**
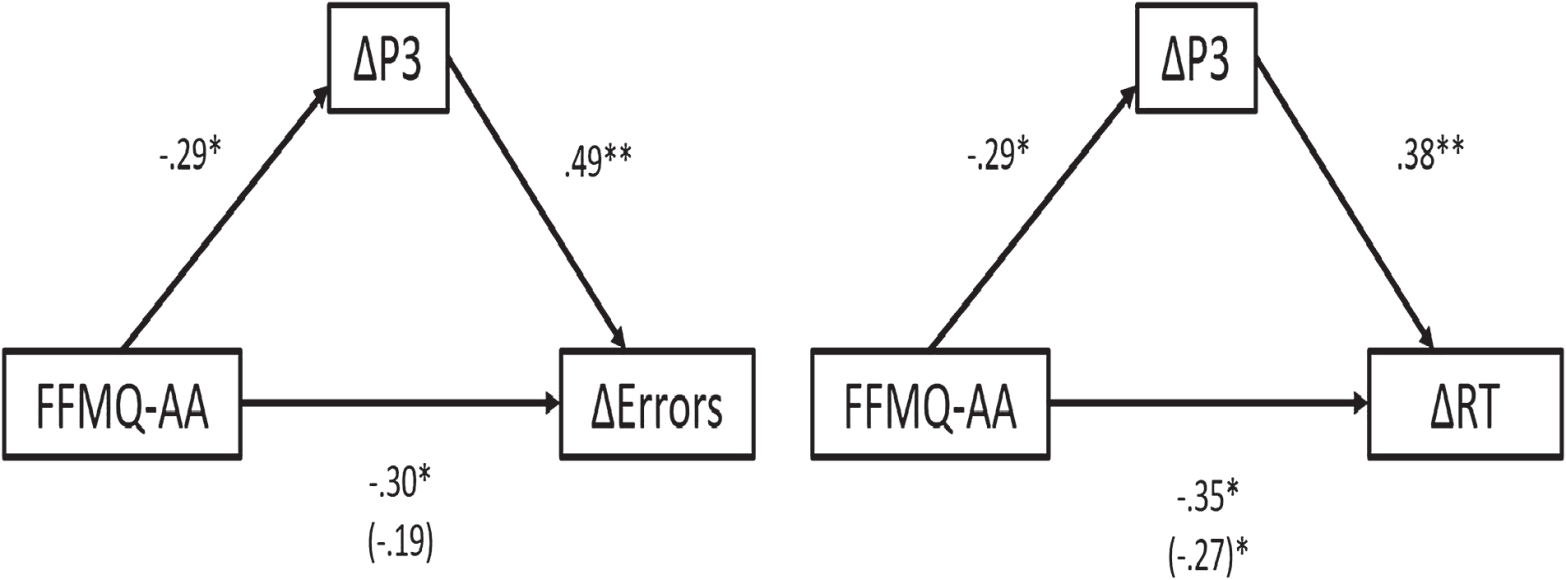
Mediational models depicting the relationship between trait mindfulness (FFMQ-AA) and the congruency (incongruent–congruent) difference in errors (Δ errors) and RT (Δ RT), as mediated by the congruency difference in P3 amplitude (ΔP3). The value in the parentheses indicates the relationship between FFMQ-AA and Δ errors and Δ RT after controlling for ΔP3 amplitude. Statistical significance is demarcated by asterisks (^*^P < 0.05, ^**^P < 0.01).

The mediation analyses revealed significant indirect effects for both models, such that smaller ΔP3 amplitudes mediated the observed negative relationship between trait mindful awareness and smaller flanker interference effects on behavioral performance (indirect effect on errors: *B* = −0.34 and 95% CI, −0.76, −0.09; indirect effect on RTs: *B* = −0.27 and 95% CI, −0.75, −0.02). See Figure 4 for a visual depiction of the models with effect sizes of the direct and indirect paths.

### Relationships between executive attention and the other facets of trait mindfulness

FFMQ-D, FFMQ-NJ and FFMQ-NR did not relate to the incongruent–congruent difference in errors (*r*s < |0.09| *P*s > 0.48), RTs (*r*s < 0.15 *P*s > 0.25) or ΔP3 (*r*s < |0.08|, *P*s > 0.56). Interestingly, FFMQ-O was positively correlated with the incongruent–congruent difference in errors (*r* = 0.26, *P* = 0.05) but not in RT (*r* = 0.09, *P* = 0.47), or ΔP3 (*r* = 0.22, *P* = 0.10). Mediation analyses were not conducted because the prerequisite conditions (i.e. causal variable significantly correlating with both outcome and mediator) were not met (Baron and Kenny, 1986).

## Discussion

Despite numerous studies, the relationship between mindfulness and executive attention remains unclear. The present study sought to clarify the nature of this relationship by examining the relationship between trait mindful awareness and behavioral and online neural measures of executive attention. Moreover, we leveraged our multimodal approach to fill a pertinent gap in the literature—namely, to delineate a specific neural mechanism through which mindfulness improves behavioral measures of executive attention.

Broadly, our findings support the presence of a positive relationship between mindfulness and executive attention. First, trait mindful awareness was related to less congruency interference on accuracy and RT, reflecting enhanced executive attention. These reductions in congruency interference effects were primarily driven by improved performance on incongruent trials. Second, analysis of the P3 revealed that higher trait mindful awareness was associated with smaller congruency interference on P3 amplitudes (i.e. ΔP3). Similar to performance accuracy, the negative association between trait mindful awareness and ΔP3 was likewise driven by smaller P3 amplitudes on incongruent, but not congruent trials. Indeed, trait mindful awareness was most related to performance and neural activity on incongruent trials—a trial type that inherently demands greater recruitment of executive attention, thereby illustrating the specificity of the relationship between mindful awareness and executive attention.

Combining ERP and behavioral measures, the mediation analyses revealed that smaller ΔP3 amplitudes mediated the reported relationships between higher trait mindful awareness and smaller congruency interference effect on both accuracy and RT. To the extent that ΔP3 reflects modulation of attention to response conflict (Clayson and Larson, 2011; Groom and Cragg, 2015), our data show that participants with high trait mindful awareness allocated fewer attentional resources to resolve the conflict between incongruent and congruent stimuli—i.e. a smaller P3 difference between the two trial types. In turn, this reduced discrepancy in attentional processing between incongruent and congruent stimuli attenuated the subsequent interference effects on performance.

Interestingly, the ancillary analyses involving the other four facets of trait mindfulness suggested that executive attention is uniquely related to trait mindful awareness. With the exception that higher trait mindful observation (FFMQ-O) related to greater congruency interference on error rate, no other relationships were detected. Given that trait mindful observation was only related to congruency interference on accuracy, but not RT or ΔP3, in conjunction with the lack of a prior theoretical foundation to anticipate this result, we can only speculate that, (i) conservatively, trait mindful observation is unlikely related to executive attention and (ii) the unique positive directionality of the relationship may reflect the extent to which the propensity to notice internal and external stimuli detracts attention from the task.

### Theoretical significance

Our findings strongly support our theoretical position that mindfulness and executive attention are overlapping constructs, insofar as trait mindful awareness, one specific facet of the broader construct of trait mindfulness, is intimately and uniquely related to executive attention. One explanatory mechanism through which trait mindful awareness attenuates ΔP3 may be via enhanced focal attention to the target. Not only have several longitudinal mindfulness training studies supported this premise (see reviews by Chiesa *et al.*, 2011; Tang *et al.*, 2015; Hӧlzel *et al.*, 2011; Smart *et al.*, 2016), but individuals with high trait mindfulness, in particular, have also been observed to exhibit increased functional activation in brain regions implicated in sustained attention (Dickenson *et al.*, 2012). By increasing focal attention on the target arrow, fewer attentional resources are allocated to the flanking arrows. This, in turn, attenuates the potency of the incorrect response representations elicited by incongruent flankers and is evidenced by smaller ΔP3 amplitudes (Clayson and Larson, 2011; Groom and Cragg, 2015). Thus, by increasing focus on the target arrow, incongruent and congruent stimuli are processed more similarly. Consequently, this reduces the congruency interference effect, culminating in improved behavioral performance of executive attention.

Our interpretation underscores the interrelated, functionally dependent nature of different attentional processes in the recruitment of executive attention (Rueda *et al.*, 2005). Furthermore, because all measures reflected congruency interference, our findings strongly suggest a specific relationship between mindfulness and executive attention (Fan *et al.*, 2003a; Fan *et al.*, 2003b). As such, calls to remove executive attention from theoretical cognitive models of mindfulness appear premature and unwarranted (Josefsson and Broberg, 2014). The current study demonstrated that trait mindful awareness and executive attention, measured across multiple levels of analysis, are related constructs that reside within the same nomological network.

### Methodological implications and future directions

The ambiguity surrounding the mindfulness–executive attention relationship from previous studies may be attributable to construct heterogeneity and method variance. On one hand, the porous boundaries between the different aspects of mindfulness introduce significant theoretical and methodological challenges (Vago and Silbersweig, 2012; Van Dam *et al.*, 2018). For example, the observed effects of mindfulness training studies are likely influenced by a confluence of factors related to other aspects of mindfulness such as trait mindfulness, state mindfulness, meditative experience and meditative style. Likewise, measures of executive attention are not homogenous. The flanker and Stroop tasks, two of the most widely employed measures, demonstrate differences in behavioral performance, ERPs (Tillman and Wiens, 2011; Riesel *et al.*, 2013), regional brain activation (Nee *et al.*, 2007) and even genetic heritability of performance (Stins *et al.*, 2004). Slight variations of the same task have also produced meaningful differences in outcomes (Salo *et al.*, 2001; Lin *et al.*, 2015). Taken together, the inconsistency among studies of mindfulness and executive attention likely speak more to construct and method variance rather than the nature of the relationship *per se*.

To this end, the current study may serve as a building block for future studies. By examining trait mindfulness in an all-female, meditation-naïve sample, we circumvented many of the aforementioned issues related to construct heterogeneity and task-related confounds (e.g. sex differences in P3 amplitudes). One obvious future direction will be to determine whether the reported effects extend to males. There is a nascent but emerging body of evidence to suggest the presence of gender differences in interest, responsivity and neural change to mindfulness interventions (Simpson *et al.*, 2007; Chen *et al.*, 2010; de Vibe *et al.*, 2013; Luders *et al.*, 2015). Given the existence of gender differences across clinical psychology (Al-Issa, 1982) and neuroscience (Cahill, 2006), consideration of sex appears pertinent toward advancing basic understanding of mindfulness and refining mindfulness-based interventions.

Although the current sample size (*n =* 60) is larger than most neurocognitive studies of mindfulness, the sample size is still modest and likely undermines the statistical power of the study. The sample size, in conjunction with the exploratory aspects of our study (e.g. testing mediation of the P3, repeated analysis involving the other facets of trait mindfulness) renders our findings preliminary. Consequently, we strongly caution against over interpretation and undue generalization of our data. It goes without saying that we encourage future studies to replicate our findings.

Future studies are also encouraged to adopt our task measures to determine the extent to which mindfulness training or other experimental manipulations of mindfulness (e.g. state mindfulness inductions) modulates executive attention in similar ways as trait mindfulness. Research on mindfulness and emotion processing involving the late positive potential (LPP) exemplifies the promise of this suggestion. Extending Brown *et al.*’s (2012) finding that high trait mindfulness relates to reduced LPP amplitude to negative emotional images, Lin *et al.* (2016) adopted their measures to uncover that brief mindfulness training, but not state mindfulness, attenuated the LPP similarly to individuals with high trait mindfulness. That is, measures that are sensitive to trait mindfulness may extend to experimental manipulations of mindfulness, creating the basis for a systematic research process that manages construct and method variance in an organized fashion. Toward this end, a recent study by Smart *et al.* (2016) offers preliminary support for the longitudinal sensitivity of the P3, finding that mindfulness training enhanced the go/no-go P3 and reduced reaction time variability in individuals with subjective cognitive decline. Although the P3 was elicited from a different task of executive function, there is nonetheless a promise that the P3 may be sensitive to extended mindfulness training and is clinically relevant. As a direct follow-up to the current study, our research team is examining whether the flanker P3 is modulated by a brief, one-time mindfulness training in a significantly larger sample (*N* = 200+). Future longitudinal studies are encouraged to examine the extent to which change in the P3 relates to change in behavioral performance across multiple tasks of executive attention. Such analyses would yield valuable data to test the theoretical propositions advanced here.

Indeed, incremental research involving standardized measures across studies may be a fruitful foundation from which to address the current challenges of the field (Van Dam *et al.*, 2018). In this vein, we view our study as a direct response to two interrelated recommendations that have rapidly emerged to the top of the *‘*prescriptive*’* agenda for improving contemplative science (Van Dam *et al.*, 2018; Tang *et al.*, 2015; Lutz *et al.*, 2015): (i) adoption of a multimodal approach so that first- (e.g. self-report) and third-person (e.g. neural) measures are analyzed to inform one another; and (ii) neuroscientific studies of mindfulness, in particular, must not solely rely on neural measures but examine the extent to which neural modulations relate to behavior. Not only does the current study demonstrate the utility of these recommendations, but it also illustrates the importance of grounding research in theory. Specifically, when examining mindfulness in relation to another construct (i.e. executive attention), it is imperative to consider the theoretical underpinnings of each construct and to identify potential areas of overlap that can inform the development of the research question, selection of measures (e.g. FFMQ-AA and P3) and generation of specific predictions. Otherwise, there is increased susceptibility to equivocate data elicited from various measures of a construct with the construct itself, possibly leading to erroneous (and often sweeping) conclusions that are theoretically untethered.

### Applied significance and current relevance

At a broader level, our findings represent empirical evidence for the mind–brain–behavior model which undergirds clinical neuroscience, demonstrating that individual differences in the propensity to engage in a particular psychological state modulated neural processing in ways that influence goal-directed behavior. Consonant with national health priorities (see NCCIH, 2018), understanding the neural mechanisms through which mindfulness confers its salutary effects on executive attention, is essential for identifying novel outcome measures, developing effective interventions and understanding the mind–brain relationship more broadly.
